# Prevalence of Sexual Initiation Before Age 13 Years Among Male Adolescents and Young Adults in the United States

**DOI:** 10.1001/jamapediatrics.2019.0458

**Published:** 2019-04-08

**Authors:** Laura D. Lindberg, Isaac Maddow-Zimet, Arik V. Marcell

**Affiliations:** 1 Guttmacher Institute, New York, New York; 2 Department of Pediatrics, School of Medicine, The Johns Hopkins University, Baltimore, Maryland; 3 Department of Population, Family, and Reproductive Health, Bloomberg School of Public Health, The Johns Hopkins University, Baltimore, Maryland

## Abstract

**Importance:**

Despite similar sexual activity rates among male and female adolescents, males are more likely to have their first sexual intercourse before age 13 years. The developmental needs and pathways to healthy trajectories for young males remain unknown.

**Objective:**

To examine the prevalence of sexual intercourse before age 13 years among male adolescents; the variation by race/ethnicity, location, and maternal educational level; and the wantedness of this first sexual experience.

**Design, Setting, and Participants:**

This cross-sectional analysis was conducted from September 2017 to June 2018, using pooled 2011, 2013, and 2015 data from the school-based Youth Risk Behavior Surveillance System (YRBSS) and the 2006 to 2015 data of males aged 15 to 24 years from the household-based National Survey of Family Growth (NSFG). The percentage of males reporting sexual onset before age 13 years was estimated using survey-weighted logistic regression to test for differences by race/ethnicity within each national survey and within metropolitan areas (for YRBSS, high school and middle school samples). Among NSFG survey respondents, differences in wantedness of first sexual intercourse by age at first sexual intercourse were examined, along with the associations between sexual initiation and socioeconomic covariates.

**Main Outcomes and Measures:**

Sexual onset before age 13 years.

**Results:**

Data from a total of 19 916 male high school students (from YRBSS) and 7739 males aged 15 to 24 years (from NSFG) were included in the analysis. The sample was largely composed of non-Hispanic white males: 8789 (57.1%) from the YRBSS and 3737 (58.0%) from the NSFG. Sexual onset before age 13 years was reported nationally by 7.6% (95% CI, 6.8%-8.4%) of male high school students and 3.6% (95% CI, 3.0%-4.2%) of males aged 15 to 24 years. The proportion of male students who reported having sexual intercourse before age 13 years varied across metropolitan sites, from 5% (95% CI, 4%-7%) in San Francisco, California, to 25% (95% CI, 23%-28%) in Memphis, Tennessee, with elevated rates among non-Hispanic black and Hispanic males in most metropolitan areas. In the NSFG data set, respondents whose mothers had a college degree or higher educational level were statistically significantly less likely (OR, 0.31; 95% CI, 0.19-0.49) to report having sexual intercourse before age 13 years compared with those whose mothers did not have a college degree. Among males who reported having their first sexual experience before age 13 years, 8.5% (95% CI, 3.8%-17.8%) described their first sexual intercourse as unwanted.

**Conclusions and Relevance:**

Rates of sexual onset before age 13 years among young males varied by race/ethnicity, location, and maternal educational level, presenting important implications for the provision of early, inclusive, and comprehensive sex education and sexual and reproductive health care to male children and adolescents.

## Key Points

**Question:**

Does the prevalence of sexual initiation before age 13 years among males in the United States vary by race/ethnicity, location, and socioeconomic status?

**Findings:**

This cross-sectional study of 19 916 male high school students and 7739 males aged 15 to 24 years found substantial variation in the rates of sexual onset before 13 years of age across metropolitan areas and by race/ethnicity, with rates as high as 28% among non-Hispanic black males in Memphis, Tennessee.

**Meaning:**

Variation in timing of sexual initiation before age 13 years may have implications for the provision of early, inclusive, and comprehensive sex education as well as sexual and reproductive health care to male children and adolescents.

## Teaser

This study examines the variations in self-reported first sexual experience among adolescent males and young male adults using data from the Youth Risk Behavior Surveillance System and National Survey of Family Growth.

## Introduction

First sexual intercourse marks an important transition in an individual’s life,^1^ and early adolescence is a critical developmental period when experimentation with sexual feelings and behaviors often begins.^2,3^ During these formative years, expectations to adhere to gender roles and norms intensify. The Centers for Disease Control and Prevention’s Youth Risk Behavior Surveillance System (YRBSS) tracks sexual intercourse before age 13 years as a core surveillance metric and finds that males are more than twice as likely as females to experience first sexual intercourse before age 13 years.^4^ Sex education guidelines recommend providing children with comprehensive sex education starting at least by kindergarten, and clinical care guidelines recommend clinicians set time alone with young patients to address confidential care inclusive of sexual health starting during early adolescence.^5,6^ However, most males start having sex before receiving sex education, and the quality of sexual health care delivery to male adolescents is poor.^7,8,9^

Estimates of young males’ transition to first sexual intercourse do not examine the prevalence of sexual activity in early adolescence across the intersecting demographics of sex, race/ethnicity, and location, likely missing important variations.^10,11,12^ A nationally representative study of sexual behavior reports it as “rare” among those 12 years and younger.^13^ Yet this conclusion may miss subgroups of males for whom sexual initiation before age 13 years is more common. Given the higher prevalence of first sexual intercourse before age 13 years among males compared with females, understanding the variation in the timing of sexual onset among adolescent males in the United States is critical to supporting their healthy sexual development.

Males’ experiences with regard to emergent manhood and sexuality are shaped by dimensions of masculinity, race/ethnicity, socioeconomic status, and location. Broad cultural scripts about masculinity and sex hold that men should start having sex early and have sex often.^14^ For young men of color, particularly black males, racist stereotypes of hypermasculinity may also contribute to expectations of early sexual initiation.^15,16,17^ Yet research highlights that males in early and middle adolescence do not necessarily follow such scripts and a later transition to first sexual intercourse may be valued.^18,19,20,21,22^ Understanding males’ wantedness of the sexual experience may be particularly important for interpreting early sexual activity.^23,24^

Aspects of adolescents’ communities may also be factors in the transition to first sexual intercourse.^25,26,27,28^ Cultural norms and values associated with masculinity may differ across communities.^29^ Some studies have found differences in the timing of first sexual intercourse between urban and rural settings.^30,31^ Even across specific urban areas, young men’s experiences may vary.

The current study examined the prevalence of sexual initiation before age 13 years among adolescent males in the United States and the variation in the timing of their sexual initiation by race/ethnicity, location, and maternal educational level and by their characterization of the wantedness of this first sexual experience. Race/ethnicity, socioeconomic status, and location are not the only factors in the timing of first sexual intercourse, but they inform the context in which these experiences and other correlates occur. We used 2 complementary large-scale representative survey systems to assess the timing of sexual onset among adolescent males in the United States, examine key sociodemographic correlates, and consider how reporting issues may affect estimates.

## Methods

This cross-sectional analysis received approval from the Guttmacher Institute Institutional Review Board, which exempted secondary data analyses that used the YRBSS and National Survey of Family Growth (NSFG) data. The study was conducted from September 2017 to June 2018.

### Data Sources

We used data from 2 cross-sectional US surveys to investigate the timing of age at first sexual intercourse among males: the YRBSS and the NSFG. The YRBSS, conducted by the Centers for Disease Control and Prevention, surveys middle school and high school students in classrooms using a paper-and-pencil, self-administered questionnaire.^32^ The national YRBSS high school sample is representative of public and private school students in grades 9 to 12. We pooled the 2011, 2013, and 2015 surveys for a total sample size of 19 916 male high school students, after excluding students with missing information on sex (<1%), age at first sexual intercourse (9%), or race/ethnicity (2%). Sample characteristics of the YRBSS and NSFG surveys are shown in Table 1.

**Table 1.** Sample Characteristics of All Male Respondents^a^

Variable	No. (Weighted %)	
	YRBSS	NSFG
All respondents	19 916 (100)	7739 (100)
Age		
<15	1950 (10.0)	NA
15-17	14 647 (74.1)	2685 (30.5)
18-19^b^	3304 (15.9)	1780 (19.3)
20-24	NA	3274 (50.2)
Race/ethnicity		
Non-Hispanic black	3148 (13.1)	1523 (15.0)
Non-Hispanic white	8789 (57.1)	3737 (58.0)
Hispanic	5914 (20.7)	1972 (20.1)
Non-Hispanic other	2065 (9.2)	507 (6.8)
Maternal educational level		
<College	UA	5793 (72.2)
≥College degree	UA	1897 (27.8)
Community type		
Urban	UA	3051 (34.8)
Suburban	UA	3529 (47.1)
Rural	UA	1159 (18.1)
Survey year: NSFG		
2006-2010	NA	4111 (50.7)
2011-2015	NA	3628 (49.3)
Survey year: YRBSS		
2011	6910 (35.4)	NA
2013	6326 (30.5)	NA
2015	6680 (34.1)	NA

Abbreviations: NA, not applicable; NSFG, National Survey of Family Growth; UA, unavailable; YRBSS, Youth Risk Behavior Surveillance System.

^a^ Data were pooled from the 2011, 2013, and 2015 YRBSS surveys and from the male respondents aged 15 to 24 years; data were pooled from the 2006 to 2010 and 2011 to 2015 continuous survey rounds of the NSFG.

^b^ The national YRBSS has only 1 top age category (18 years or older). Thus, this category potentially includes a small number of male high school students aged 20 years or older.

To examine the variation across geographic settings, we used representative YRBSS data from 8 middle school and 17 high school metropolitan areas. We used all publicly available YRBSS metropolitan area surveys and obtained permission to use some nonpublic YRBSS data from additional areas that had weighted data for at least 2 of the 3 survey years. In these high school samples, missingness on age at first sexual intercourse ranged from 8% to 26%. No national middle school YRBSS is conducted, but we included available metropolitan-area middle school data; these data were collected from younger students, potentially limiting recall bias in reporting age at first sexual intercourse.

The NSFG is a periodic national probability survey of the noninstitutionalized population of females and males aged 15 to 44 years in the United States.^33,34,35^ To increase our sample size while minimizing the length of retrospective recall, we examined pooled data from the 2006 to 2010 and 2011 to 2015 continuous survey rounds and limited our analyses to 7739 males aged 15 to 24 years at the time of interview. The NSFG is administered through face-to-face interviews, and more sensitive questions are asked through the ACASI (audio computer-assisted self-interview) method.

### Measures

#### Age at First Sexual Intercourse

The YRBSS and NSFG surveys asked the age at which respondents first had sexual intercourse. The YRBSS asked age at first sexual intercourse without specifying the sex of the respondent’s partner, whereas the NSFG asked the timing of first heterosexual sexual intercourse. For both surveys, the answer to had sexual intercourse before age 13 years was dichotomized, with 0 indicating no and 1 indicating yes; respondents who had not had sex by the time of interview were coded as 0’s.

#### Wantedness of First Sexual Intercourse

The NSFG asked this question of all sexually experienced male respondents aged 18 years or older: Think back to the very first time you had vaginal intercourse with a female. Which would you say comes closest to describing how much you wanted that first vaginal intercourse to happen? Response choices were as follows: “I really didn’t want it to happen at the time,” “I had mixed feelings—part of me wanted it to happen at the time and part of me didn’t,” and “I really wanted it to happen at the time.”

#### Demographic and Survey Characteristics

The surveys included comparable measures of race/ethnicity (non-Hispanic white, non-Hispanic black, non-Hispanic other, and Hispanic). Non-Hispanic other comprised Asian, American Indian, Alaskan Native, and Hawaiian and Pacific Islander males, but small sample sizes did not allow for separate analyses of each group. The national YRBSS did not include other sociodemographic covariates or details on location. From the NSFG data, we examined maternal educational level (less than college, or completed college or more) as a proxy for socioeconomic status, and community type (urban, suburban, or rural). We also constructed indicators for survey wave. To identify possible retrospective reporting bias, we used respondents’ integer age at interview (NSFG) and grade in school (YRBSS).

### Statistical Analysis

We estimated the percentage of male high school students (national YRBSS) and males aged 15 to 24 years (NSFG) reporting sexual onset before age 13 years, testing for within-survey differences by race/ethnicity using unadjusted logistic regression. We examined differentials by metropolitan area using the YRBSS metropolitan samples, and we tested for differences by race/ethnicity within each site, controlling for grade and survey year to adjust for differences in sample composition. To account for censoring in the middle school data, given that some respondents were not yet 13 years of age, we estimated proportions from a Cox proportional hazards regression model (adjusted for grade and survey year); significance tests are for differences in site-specific relative hazards of early sexual onset between race/ethnicity groups.

Using NSFG data, we estimated multivariable logistic regression models of sexual onset before age 13 years as a function of race/ethnicity, maternal educational level, community type, survey year, and age at interview. Then, we estimated probabilities of sexual onset before age 13 years by race/ethnicity and maternal educational level, after adding an interaction term between those 2 variables to the model. We also examined differences by age at first sexual intercourse in wantedness of first sexual intercourse (among respondents aged 18-24 years in the NSFG).

To further compare differences in estimates between the YRBSS and NSFG, we conducted sensitivity analyses exploring alternate specifications in the NSFG, including using ACASI measures of age at first sexual intercourse, limiting the sample to those currently aged 15 to 19 years and in school and dropping all imputed data on age at first sexual intercourse. To test if differences persisted across age, we produced Kaplan-Meier life table estimates of age at first sexual intercourse for both data sets.

All analyses were weighted. All *P* values were calculated using 2-tailed Wald tests, with SEs adjusted for the complex survey design of each data source using Stata, version 15.1 (StataCorp).^36^ Statistical significance was determined using α = .05.

## Results

In total, this study analyzed 19 916 male high school students from the YRBSS data set and 7739 males aged 15 to 24 years from the NSFG data set. The sample was largely composed of non-Hispanic white males: 8789 (57.1%) from the YRBSS and 3737 (58.0%) from the NSFG.

### Prevalence of Early Sexual Onset by Race/Ethnicity

The YRBSS and NSFG national data show different levels of sexual activity before age 13 years, but similar differentials by race/ethnicity. Sexual onset before age 13 years was reported by 7.6% (95% CI, 6.8%-8.4%) of male students in grades 9 to 12 in the YRBSS and 3.6% (95% CI, 3.0%-4.2%) of males aged 15 to 24 years in the NSFG (Table 2; results by survey year are available in eTable 1 in the Supplement). In both surveys, reports of early first sexual intercourse were statistically significantly higher among non-Hispanic black males than other racial/ethnic groups.

**Table 2.** Weighted Percentage of Male Students Reporting Their First Sexual Intercourse Before Age 13 Years, by Race/Ethnicity^a^

Race/Ethnicity	YRBSS		*P* Value	NSFG		*P* Value
	Total Unweighted Sample Size	% (95% CI)		Total Unweighted Sample Size	% (95% CI)	
Total	19 916	7.6 (6.8-8.4)	NA	7739	3.6 (3.0-4.2)	NA
Non-Hispanic black [reference]	3148	19.0 (16.7-21.3)	NA	1523	10.5 (8.4-12.5)	NA
Non-Hispanic white	8789	4.4 (3.8-5.0)	<.001	3737	2.2 (1.5-2.9)	<.001
Hispanic	5914	9.0 (7.8-10.1)	<.001	1972	3.4 (2.3-4.5)	<.001
Non-Hispanic other	2065	7.8 (5.8-9.7)	<.001	507	1.6 (0.3-2.9)	<.001

Abbreviations: NA, not applicable; NSFG, National Survey of Family Growth; YRBSS, Youth Risk Behavior Surveillance System.

^a^ Data were pooled from the 2011, 2013, and 2015 YRBSS surveys and from the male respondents aged 15 to 24 years; data were pooled from the 2006 to 2010 and 2011 to 2015 continuous survey rounds of the NSFG.

### Prevalence of Early Sexual Onset by Metropolitan Area and Race/Ethnicity

Across the 15 metropolitan sites with available YRBSS high school data, the proportion of male students who reported having their first sexual intercourse before age 13 years varied widely, from 5% (95% CI, 4%-7%) in San Francisco, California, to 25% (95% CI, 23%-28%) in Memphis, Tennessee, after controlling for grade or year of survey (Table 3). In most areas, higher proportions of non-Hispanic black male students reported having sexual intercourse before age 13 years compared with those in other racial/ethnic groups. Prevalence by race/ethnicity varied across metropolitan areas, ranging from 12% (95% CI, 7%-17%) in Seattle, Washington, to 28% (95% CI, 25%-31%) in Memphis among non-Hispanic black males; from 6% (95% CI, 4%-7%) in Los Angeles, California, to 17% in Seattle (95% CI, 10%-24%) and in Memphis (95% CI, 8%-27%) among Hispanic males; from 2% (95% CI, 0%-3%) in Charlotte-Mecklenburg, North Carolina, to 10% (95% CI, 3%-16%) in Chicago, Illinois, among non-Hispanic white males; and from 2% (95% CI, 1%-3%) in San Francisco to 17% (95% CI, 8%-27%) in Chicago among male students in the non-Hispanic other race/ethnicity category.

**Table 3.** Estimated Proportion of Male High School Students Reporting Their First Sexual Intercourse Before Age 13 Years, by Race/Ethnicity and Location, Adjusted for Grade and Survey Year^a^

Location	Total Unweighted Sample Size	% (95% CI)			*P* Value	Hispanic, % (95% CI)	*P* Value	Non-Hispanic Other, % (95% CI)	*P* Value
		Total	Non-Hispanic Black	Non-Hispanic White					
Memphis, TN	1089	25 (23-28)	28 (25-31)	3 (0-6)	<.001	17 (8-27)	.08	16 (7-26)	.06
Milwaukee, WI	1076	19 (16-22)	25 (20-29)	5 (0-11)	.002	15 (10-20)	.006	11 (6-17)	.003
Chicago, IL	1243	18 (15-21)	29 (23-35)	10 (3-16)	.002	11 (8-14)	<.001	17 (8-27)	.01
Boston, MA	1507	14 (12-17)	17 (12-22)	9 (4-13)	.03	16 (12-19)	.67	8 (4-12)	.002
Duval County, FL	3770	13 (11-14)	20 (18-23)	6 (4-7)	<.001	15 (11-18)	.03	10 (7-12)	<.001
Houston, TX	2507	12 (11-14)	22 (18-27)	6 (2-10)	<.001	10 (8-11)	<.001	5 (2-9)	<.001
Miami-Dade County, FL	3146	11 (10-13)	20 (17-23)	6 (2-11)	<.001	9 (8-11)	<.001	15 (7-23)	.35
Charlotte-Mecklenburg, NC	1293	11 (8-13)	20 (15-24)	2 (0-3)	<.001	10 (7-14)	<.001	11 (6-16)	.01
Palm Beach County, FL	1650	10 (9-12)	18 (14-22)	5 (3-7)	<.001	12 (9-16)	.03	12 (7-17)	.08
Broward County, FL	1840	10 (9-12)	19 (15-22)	3 (1-4)	<.001	9 (7-11)	<.001	8 (3-13)	.02
Orange County, FL	1986	10 (9-12)	20 (15-25)	4 (2-6)	<.001	10 (8-13)	<.001	8 (4-12)	<.001
New York City, NY	10 228	9 (8-10)	15 (12-17)	3 (2-4)	<.001	11 (10-12)	.005	3 (2-3)	<.001
San Bernardino, CA	1204	9 (7-11)	14 (9-20)	9 (3-14)	.14	9 (7-11)	.03	4 (0-8)	.01
San Diego, CA	2381	7 (6-9)	13 (8-18)	5 (3-7)	.002	9 (7-11)	.08	4 (2-6)	<.001
Los Angeles, CA	1393	7 (5-8)	14 (3-25)	5 (1-9)	.06	6 (4-7)	.03	4 (2-7)	.02
Seattle, WA	1441	6 (5-8)	12 (7-17)	3 (2-5)	<.001	17 (10-24)	.23	3 (1-4)	<.001
San Francisco, CA	2637	5 (4-7)	27 (17-37)	3 (0-6)	<.001	10 (8-13)	<.001	2 (1-3)	<.001

^a^ Data were pooled from the 2011, 2013, and 2015 Youth Risk Behavior Surveillance System surveys. To be included in the sample, sites needed to have data available for at least 2 of the 3 survey years. Proportions were estimated from a logistic regression model controlling for grade and survey year; race/ethnicity-specific proportions were estimated from models with race/ethnicity by site interaction terms included as covariates. All significance tests used non-Hispanic black as a reference group.

In the YRBSS middle school data (eTable 2 in the Supplement), reporting of having sexual intercourse before age 13 years had similar differentials by race/ethnicity as the high school reports. However, in almost all metropolitan areas, the proportion of students who reported having intercourse before age 13 years was higher in the middle school sample compared with the corresponding high school sample.

### Multivariable Models of Early Sexual Onset

Analyses of the NSFG data revealed that non-Hispanic white males (odds ratio [OR], 0.21; 95% CI, 0.13-0.32), Hispanic males (OR, 0.27; 95% CI, 0.18-0.4), and non-Hispanic other males (OR, 0.16; 95% CI, 0.06-0.38) were statistically significantly less likely to report having their first sexual intercourse before age 13 years than non-Hispanic black males, after adjusting for sociodemographic variables (Table 4). Respondents whose mothers had a college degree or higher educational level were statistically significantly less likely (OR, 0.31; 95% CI, 0.19-0.49) to report having sexual intercourse before age 13 years compared with those whose mothers did not have a college degree. No statistically significant differences were found by community type or by survey wave. Respondents’ age at interview was positively associated with reporting having sexual intercourse before age 13 years. The absolute difference was modest, however; we estimated that 3% of 16-year-old and 4% of 20-year-old respondents would report their sexual activity before 13 years of age.

**Table 4.** Multivariable Logistic Regression of the Association Between Sexual Intercourse Before Age 13 Years and Various Demographic Characteristics

Variable	OR (95% CI)	*P* Value
Race/ethnicity		
Non-Hispanic black	1 [Reference]	
Non-Hispanic white	0.21 (0.13-0.32)	<.001
Hispanic	0.27 (0.18-0.4)	<.001
Non-Hispanic other	0.16 (0.06-0.38)	<.001
Maternal educational level		
<College	1 [Reference]	
≥College degree	0.31 (0.19-0.49)	<.001
Community type		
Urban	0.87 (0.52-1.45)	.58
Suburban	0.76 (0.45-1.26)	.29
Rural	1 [Reference]	
Survey year		
2006-2010	1 [Reference]	
2011-2015	1.16 (0.81-1.67)	.40
Age of respondent at interview	1.09 (1.03-1.14)	.001
Estimated probabilities for race/ethnicity by maternal educational level, % (95% CI)^a^		
Non-Hispanic black: <college [reference]	12 (9-14)	
Non-Hispanic black: ≥college degree	6 (2-10)	.03
Non-Hispanic white: <college	3 (2-4)	<.001
Non-Hispanic white: ≥college degree	0 (0-1)	<.001
Hispanic: <college	4 (2-5)	<.001
Hispanic: ≥college degree	2 (0-3)	<.001
Non-Hispanic other: <college	2 (0-4)	<.001
Non-Hispanic other: ≥college degree	1 (0-2)	<.001

Abbreviations: NSFG, National Survey of Family Growth; OR, odds ratio.

^a^ Estimated from additional model, including interaction term between race/ethnicity and maternal educational level (eTable 3 in the Supplement). Data from the male respondents aged 15 to 24 years pooled from the 2006 to 2010 and 2011 to 2015 continuous survey rounds of the NSFG.

After adding to the model interaction terms between race/ethnicity and maternal educational level (eTable 3 in the Supplement), we estimated that 12% (95% CI, 9%-14%) of non-Hispanic black males whose mothers did not have a college degree would report having their first intercourse before age 13 years (Table 4); this probability is statistically significantly higher than for males in any other combination of race/ethnicity and maternal educational level, which ranged from 0% (95% CI, 0%-1%) for non-Hispanic white males whose mothers had a college degree or more to 6% (95% CI, 2%-10%) for non-Hispanic black males whose mothers had a college degree or more.

#### Wantedness of First Sexual Intercourse by Age of Sexual Onset

Among respondents aged 18 to 24 years who reported having their first sexual intercourse before age 13 years, 8.5% (95% CI, 3.8%-17.8%) characterized it as unwanted, 37.0% (95% CI, 28.0%-47.0%) had mixed feelings about it, and 54.6% (95% CI, 44.7%-64.1%) described it as wanted (Figure). These distributions were not statistically significantly different from those among males who had sexual intercourse at age 13 years or later: 5.4% reported it as unwanted, 31.3% had mixed feelings, and 63.3% described it as wanted. Most of the respondents who reported having sex before age 13 years described their first sexual partner as a friend (eTable 4 in the Supplement).

**Figure.**
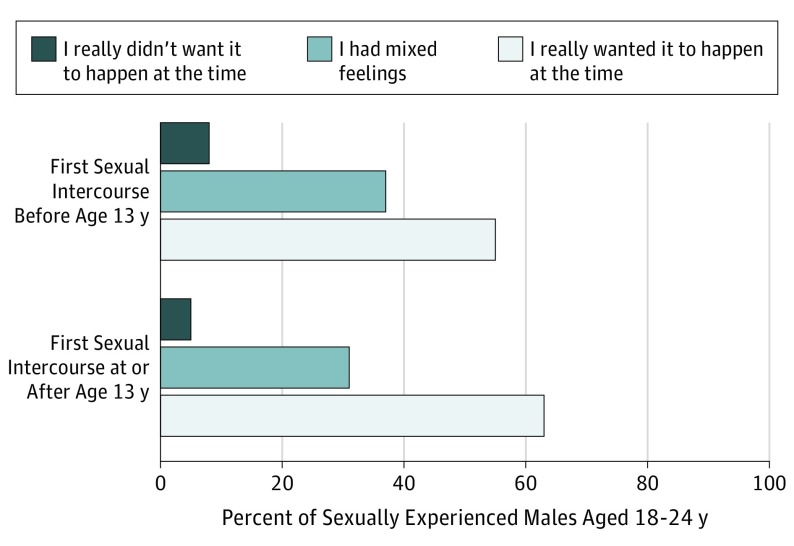
Percent Distribution of Wantedness of First Sexual Intercourse Among Sexually Experienced Male Respondents Aged 18 to 24 Years, by Age at First Sexual Intercourse Data were pooled from the 2006 to 2010 and 2011 to 2015 continuous survey rounds of the National Survey of Family Growth.

#### Sensitivity Analysis

None of the sensitivity adjustments to the NSFG data (using ACASI measures of age at first sexual intercourse, limiting the sample to those currently aged 15 to 19 years and in school, or dropping all imputed data on age at first sexual intercourse) narrowed the gap between the NSFG and YRBSS prevalence estimates (eTable 5 in the Supplement). Kaplan-Meier life table estimates of age at first sexual intercourse between the NSFG and the YRBSS data sets showed persistent differences across age (eFigure in the Supplement).

## Discussion

Drawing on representative surveys, we found that reported rates of sexual onset before age 13 years among adolescent males in the United States varied substantially by race/ethnicity, location, and maternal educational level. Although males in the YRBSS reported a higher prevalence of sex before 13 years of age than males in the NSFG, the differentials by race/ethnicity in each survey were extremely similar. The NSFG data also showed substantial differentials by maternal educational level, even after controlling for race/ethnicity. These findings underscore the need for providing comprehensive sex education that is culturally informed and inclusive before an individual’s first sexual encounter and ensuring that health care practitioners discuss sex with their male patients starting during middle school years or earlier.

This study extends previous findings that showed a high prevalence of sexual activity before age 13 years, particularly among males of color, and highlights substantial variation across metropolitan areas.^4,13,37^ These findings reinforce that males’ identities and community contexts are associated with their experiences. Other studies have found that age at first sexual intercourse is associated with identifiable systemic barriers in communities, such as racial segregation^25^ and neighborhood disadvantage.^28,30^ Adolescent males’ experiences of emerging sexuality are informed by their social context, which is often defined by location. To this end, investments at the local level will be critical to support and promote youth development generally and healthy sexual development specifically.

Some of the reported first sexual experiences were characterized as unwanted and mixed feelings were common, but more than half reported their experience as wanted. This finding underscores the need to include young men’s views when identifying and interpreting their sexual and developmental trajectories. However, wantedness of early sexual intercourse among male adolescents may also represent efforts to conform to traditional cultural expectations of masculinity and may not on its own represent healthy sexual choices. Health education and counseling can create opportunities for young males, their families, and communities to discuss healthy sexuality, including topics of consent, coercion, and development of sexual expression.^3,38^ Systems of care are also needed to support and treat young males who have experienced unwanted sexual encounter.

Normative masculinity values may be a factor in reporting of age at first sexual experience, explaining some of the observed race/ethnicity differentials. Studies have found inconsistent reporting of first sexual intercourse among black males, which is suggestive of overreporting of younger ages at first sexual intercourse.^39^ Social pressures for such reporting may be a particular factor in responses to school-based surveys, which may partially explain the higher rates in the YRBSS data compared with rates in the NSFG data.^40^ Other survey differences, including sampling frames, survey modes, and survey measures, may also explain some of these differences.^41^

Although race/ethnicity and gender-specific norms may be a factor in reporting of age at first sexual intercourse, such norms in and of themselves are relevant to adolescent males’ actual experiences and development. For example, black children, particularly males, often are viewed as more adult than white children, without the need for protection afforded to others.^42^ Health care practitioners should work together with parents or guardians and their sons during late middle childhood and early adolescence to identify and respond to the normative pressures around masculinity.^43^

Early sexual onset has been associated with increased prevalence of negative physical and mental health outcomes.^44^ The causal role of age at first sexual intercourse is unclear, however, and associations may be driven by unmeasured confounding factors such as childhood sexual abuse, pubertal timing, or parental engagement. Emerging research suggests that the context of sexual initiation may have greater implications for sexual health than age alone.^45^ Our focus on sexual onset before age 13 years recognizes an important developmental and social marker. Variation exists in when cognitive, emotional, and physical development milestones are reached, and other studies have used different chronological cutoffs in their investigation of early first sexual intercourse,^44,46^ but there is no particular biological age at which individuals are uniformly ready for this transition. Policy approaches that aim to delay sexual onset may focus on the wrong drivers of behavior and may risk causing harm through stigmatization of and barriers to sex education and sexual health services.^47^

Healthy People 2020 calls for providing sex education to adolescents before age 18 years.^48^ Ideally, this education should begin before an adolescent first has sexual intercourse, but fewer than half of all teenaged males receive formal instruction on birth control before their first sexual encounter.^7^ In addition, parents are less likely to talk with their sons than daughters about many sexual health topics.^39^ Health care practitioners should expand how they fill these gaps during early adolescence and late middle childhood. Although the Bright Futures guidelines for early adolescence discuss screening and anticipatory guidance for sexual activity, as well as the importance of individual time with a clinician, its guideline for middle childhood is focused on sexual abuse and offers less guidance on the importance of visit time alone.^49^ Our finding that substantial shares of male adolescents have their first sexual intercourse before age 13 years underscores the need to provide comprehensive sex education and sexual and reproductive health care that start early and are developmentally appropriate for children’s age.^6^ Greater attention also needs to be given to providing sex education that is culturally informed and inclusive.^50^

### Limitations

This study has several limitations. Age at first sexual intercourse is only 1 indicator of sexuality; efforts to understand the trajectories of sexual development should consider behaviors beyond vaginal-penile intercourse.^51^ Furthermore, the available measures are fairly blunt constructs of the intersection of sex, race/ethnicity, socioeconomic status, and location and likely miss further nuance and differentiation. In addition, the increased reporting of earlier occurrence of first sexual intercourse in the middle school data compared with the high school YRBSS and by age in the NSFG is suggestive of reporting bias associated with age or length of recall. The study’s use of multiple high-quality surveys to triangulate reports offsets this limitation.

## Conclusions

Rates of sexual onset before age 13 years among young males in the United States varied widely by race/ethnicity, location, and maternal educational level, with higher rates among non-Hispanic black males in most metropolitan areas. These findings may have major implications for the timing of sex education and sexual and reproductive health care. Helping parents or guardians, schools, and communities support male adolescents’ healthy sexual development should be a priority. Health care practitioners must recognize and address all of the developmental needs and pathways to healthy trajectories for young males.

## Notes

Supplement. **eTable 1.** Percent of Male Students Reporting Their First Sexual Intercourse Before Age 13 Years, 2011, 2013 and 2015 YRBS; Percent of Young Men Aged 15-24 Reporting Their First Sexual Intercourse Before Age 13 Years, 2006-2010 and 2011-2015 NSFG **eTable 2.** Predicted Proportion of Male Middle School Students Reporting Their First Sexual Intercourse Before Age 13 Years, by Race/Ethnicity and Location **eTable 3.** Full Multivariable Logistic Regression Model of the Association Between First Sexual Intercourse Before Age 13 Years and Various Demographic Characteristics, Used to Produce Predicted Probabilities in eTable 2 **eTable 4.** Relationship with First Sexual Partner, Among Men Aged 15-24 Reporting Their First Sexual Intercourse Before Age 13 Years, Pooled 2006-2010 and 2011-2015 NSFG **eTable 5.** Sensitivity Analyses of Percent of Males Aged 15-24 Reporting Their First Sexual Intercourse Before Age 13 Years, Pooled 2006-10 and 2011-15 NSFG; According to Race/Ethnicity and Sensitivity Specification **eFigure.** Kaplan-Meier Failure Curve of Age at First Sex Among Male 15-24 Year Olds, Pooled NSFG 2006-10 and 2011-15, and Male Students, Pooled YRBS 2011-2015
