# Supplementary material for: Prevalence of Sexual Initiation Before Age 13 Years Among Male Adolescents and Young Adults in the United States
Source: JAMA Pediatr. 2019 Apr 8;173(6):553–60. doi: 10.1001/jamapediatrics.2019.0458 (PMC6547075; doi:10.1001/jamapediatrics.2019.0458)
Supplement: Supplement. — eTable 1. Percent of Male Students Reporting Their First Sexual Intercourse Before Age 13 Years, 2011, 2013 and 2015 YRBS; Percent of Young Men Aged 15-24 Reporting Their First Sexual Intercourse Before Age 13 Years, 2006-2010 and 2011-2015 NSFG eTable 2. Predicted Proportion of Male Middle School Students Reporting Their First Sexual Intercourse Before Age 13 Years, by Race/Ethnicity and Location eTable 3. Full Multivariable Logistic Regression Model of the Association Between First Sexual Intercourse Before Age 13 Years and Various Demographic Characteristics, Used to Produce Predicted Probabilities in eTable 2 eTable 4. Relationship with First Sexual Partner, Among Men Aged 15-24 Reporting Their First Sexual Intercourse Before Age 13 Years, Pooled 2006-2010 and 2011-2015 NSFG eTable 5. Sensitivity Analyses of Percent of Males Aged 15-24 Reporting Their First Sexual Intercourse Before Age 13 Years, Pooled 2006-10 and 2011-15 NSFG; According to Race/Ethnicity and Sensitivity Specification eFigure. Kaplan-Meier Failure Curve of Age at First Sex Among Male 15-24 Year Olds, Pooled NSFG 2006-10 and 2011-15, and Male Students, Pooled YRBS 2011-2015 [file jamapediatr-173-553-s001.pdf]

Supplementary Online Content

Lindberg LD, Maddow-Zimet I, Marcell AV. Prevalence of sexual initiation before age 13 years among male adolescents and young adults in the United States. *JAMA Pediatr.* Published online April 8, 2019. doi:10.1001/jamapediatrics.2019.0458

eTable 1. Percent of Male Students Reporting Their First Sexual Intercourse Before Age 13 Years, 2011, 2013 and 2015 YRBS; Percent of Young Men Aged 15-24 Reporting Their First Sexual Intercourse Before Age 13 Years, 2006-2010 and 2011-2015 NSFG

eTable 2. Predicted Proportion of Male Middle School Students Reporting Their First Sexual Intercourse Before Age 13 Years, by Race/Ethnicity and Location

eTable 3. Full Multivariable Logistic Regression Model of the Association Between First Sexual Intercourse Before Age 13 Years and Various Demographic Characteristics, Used to Produce Predicted Probabilities in Table 4

eTable 4. Relationship with First Sexual Partner, Among Men Aged 15-24 Reporting Their First Sexual Intercourse Before Age 13 Years, Pooled 2006-2010 and 2011-2015 NSFG

eTable 5. Sensitivity Analyses of Percent of Males Aged 15-24 Reporting Their First Sexual Intercourse Before Age 13 Years, Pooled 2006-10 and 2011-15 NSFG; According to Race/Ethnicity and Sensitivity Specification

eFigure. Kaplan-Meier Failure Curve of Age at First Sex Among Male 15-24 Year Olds, Pooled NSFG 2006-10 and 2011-15, and Male Students, Pooled YRBS 2011-2015

This supplementary material has been provided by the authors to give readers additional information about their work.

© 2019 Lindberg et al. *JAMA Pediatrics*.

eTable 1. Percent of Male Students Reporting Their First Sexual Intercourse Before Age 13 Years, 2011, 2013 and 2015 YRBS; Percent of Young Men Aged 15-24 Reporting Their First Sexual Intercourse Before Age 13 Years, 2006-2010 and 2011-2015 NSFG

Survey year	%	95% CI
**YRBS**		
2011	*9%*	*(8%-10%)*
2013	*8%*	*(7%-10%)*
2015	*6%*	*(4%-7%)*
**NSFG**		
2006-2010	3%	*(3%-4%)*
2011-2015	4%	*(3%-5%)*

© 2019 Lindberg et al. *JAMA Pediatrics*.

eTable 2. Predicted Proportion of Male Middle School Students Reporting Their First Sexual Intercourse Before Age 13 Years, by Race/Ethnicity and Location[a]

Location	*Total unweighted sample size*	Total *(95% CI)*	Non-Hispanic black *(95% CI)*	Non-Hispanic white *(95% CI)*	p-value	Hispanic *(95% CI)*	p-value	Non-Hispanic other *(95% CI)*	p-value
Memphis, TN	*1,098*	29% *(19%-38%)*	32% *(21%-43%)*	*na*		12% *(4%-20%)*	0.002	*na*	
Milwaukee, WI	*1,744*	27% *(18%-36%)*	40% *(25%-55%)*	7% *(3%-12%)*	<.001	11% *(6%-16%)*	<.001	18% *(9%-27%)*	<.001
Duval County, FL	*5,639*	17% *(12%-23%)*	27% *(18%-36%)*	9% *(6%-12%)*	<.001	20% *(13%-27%)*	0.008	16% *(10%-22%)*	<.001
Chicago, IL	*1,144*	15% *(10%-20%)*	27% *(17%-37%)*	*na*		8% *(4%-11%)*	<.001	*na*	
Palm Beach, FL	*2,407*	12% *(9%-16%)*	26% *(17%-35%)*	4% *(2%-6%)*	<.001	13% *(8%-18%)*	<.001	10% *(5%-16%)*	<.001
Broward County, FL	*1,226*	12% *(10%-14%)*	20% *(17%-23%)*	5% *(2%-8%)*	<.001	10% *(5%-15%)*	0.004	14% *(6%-22%)*	0.19
Orange County, FL	*2,218*	11% *(8%-14%)*	24% *(15%-32%)*	4% *(2%-6%)*	<.001	10% *(6%-14%)*	<.001	9% *(3%-15%)*	0.002
San Francisco, CA	*2,972*	6% *(4%- 9%)*	30% *(17%-44%)*	6% *(2%-9%)*	<.001	10% *(6%-14%)*	<.001	2% *(1%-3%)*	<.001

Note: Proportions are predicted cumulative hazards from hazard model controlling for grade and survey year. All race specific cumulative hazards predicted from models with race X site interaction terms included as covariates. All significance tests use non-Hispanic black as a reference group.

[a] In order to be included in the sample, sites needed to have data available for at least two of the three survey years.

© 2019 Lindberg et al. *JAMA Pediatrics*.

eTable 3. Full Multivariable Logistic Regression Model of the Association Between First Sexual Intercourse Before Age 13 Years and Various Demographic Characteristics, Used to Produce Predicted Probabilities in eTable 2

	OR	95% CI	p-value
Race/ethnicity			
Non-Hispanic black	1.00		
Non-Hispanic white	0.23	*(0.15-0.36)*	<.001
Hispanic	0.28	*(0.18-0.42)*	<.001
Non-Hispanic other	0.16	*(0.06-0.43)*	<.001
Mother's education			
Less than college	1.00		
College or more	0.49	*(0.26-0.94)*	0.03
Community type			
Urban	0.89	*(0.53-1.49)*	0.65
Suburban	0.76	*(0.46-1.28)*	0.31
Rural (ref)	1.00		
Survey year			
2006-2010 (ref)	1.00		
2011-2015	1.16	*(0.81-1.66)*	0.42
Age of respondent at interview	1.08	*(1.03-1.14)*	<.001
Interaction of race/ethnicity and mother's education			

© 2019 Lindberg et al. *JAMA Pediatrics*.

Non-Hispanic white X College or more	0.32	*(0.1-1.05)*	0.06
Hispanic X College or more	0.98	*(0.34-2.82)*	0.97
Non-Hispanic other X College or more	0.69	*(0.12-4.05)*	0.68

eTable 4. Relationship with First Sexual Partner, Among Men Aged 15-24 Reporting Their First Sexual Intercourse Before Age 13 Years, Pooled 2006-2010 and 2011-2015 NSFG

**Relationship type**	%	95% CI
Just friends	54%	*(47%-60%)*
Going out	28%	*(22%-35%)*
“Had just met her”/something else	18%	*(14%-24%)*

© 2019 Lindberg et al. *JAMA Pediatrics*.

eTable 5. Sensitivity Analyses of Percent of Males Aged 15-24 Reporting Their First Sexual Intercourse Before Age 13 Years, Pooled 2006-10 and 2011-15 NSFG; According to Race/Ethnicity and Sensitivity Specification

	ACASI reports of age at first sex				Aged 15-19 currently in school				Respondents with imputed age at first sex excluded			
Race/ethnicity	*N*[a]	%	95% CI	p-value	*N*[a]	%	95% CI	p-value	*N*[a]	%	95% CI	p-value
Total	*7,739*	4%	*(3%-4%)*		*3,696*	4%	*(3%-4%)*		*6,736*	4%	*(3%-4%)*	
Non-Hispanic black (ref)	*1,523*	9%	*(7%-12%)*		*749*	7%	*(5%-9%)*		*1,239*	11%	*(8%-13%)*	
Non-Hispanic white	*3,737*	2%	*(1%-2%)*	<.001	*1,724*	1%	*(1%-2%)*	<.001	*3,356*	2%	*(1%-3%)*	<.001
Hispanic	*1,972*	3%	*(1%-4%)*	<.001	*964*	2%	*(1%-3%)*	<.001	*1,677*	3%	*(2%-5%)*	<.001
Non-Hispanic other	*507*	1%	*(0%-2%)*	<.001	*259*	1%	*(0%-3%)*	0.008	*464*	2%	*(0%-3%)*	<.001

[a]Total unweighted sample size

© 2019 Lindberg et al. *JAMA Pediatrics*.

eFigure 1. Kaplan-Meier failure curve of age at first sex among male 15-24 year olds, pooled NSFG 2006-10 and 2011-15, and male students, pooled YRBS 2011-2015

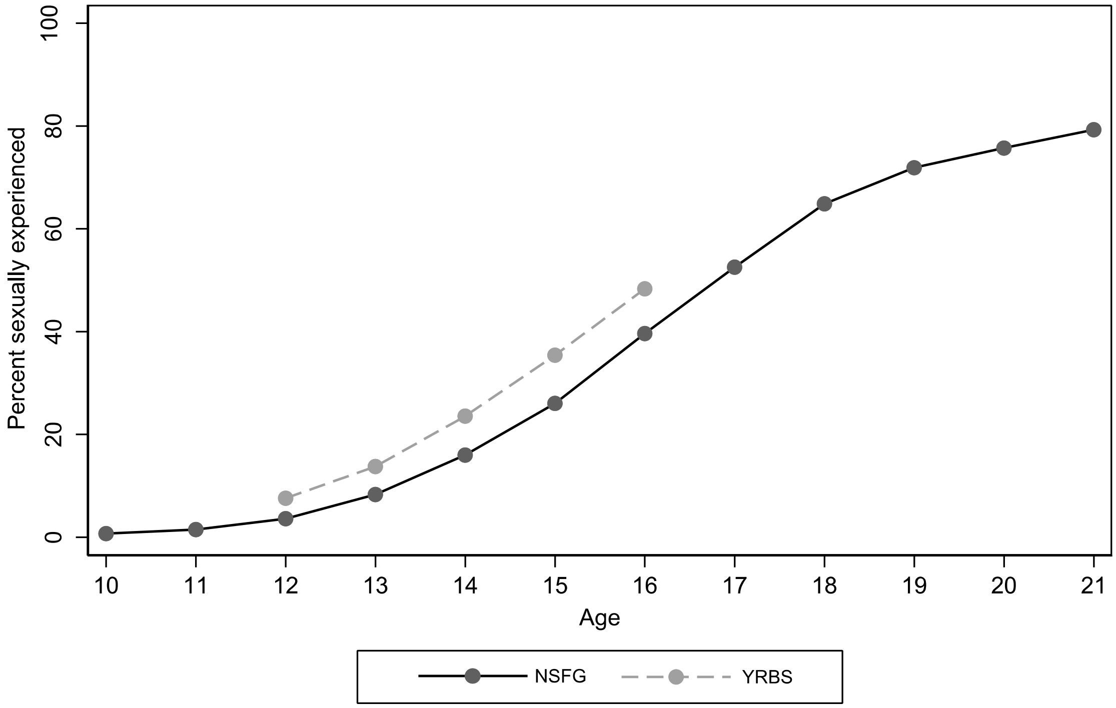

© 2019 Lindberg et al. *JAMA Pediatrics*.
